# Design and Early Implementation Successes and Challenges of a Pharmacogenetics Consult Clinic

**DOI:** 10.3390/jcm9072274

**Published:** 2020-07-17

**Authors:** Meghan J. Arwood, Eric A. Dietrich, Benjamin Q. Duong, D. Max Smith, Kelsey Cook, Amanda Elchynski, Eric I. Rosenberg, Katherine N. Huber, Ying L. Nagoshi, Ashleigh Wright, Jeffrey T. Budd, Neal P. Holland, Edlira Maska, Danielle Panna, Amanda R. Elsey, Larisa H. Cavallari, Kristin Wiisanen, Julie A. Johnson, John G. Gums

**Affiliations:** 1 Department of Pharmacotherapy and Translational Research, College of Pharmacy, University of Florida, 1345 Center Dr, Gainesville, FL 32603, USA; dietrich@cop.ufl.edu (E.A.D.); Benjamin.Duong@nemours.org (B.Q.D.); Max.Smith@medstar.net (D.M.S.); kelsey.cook@ufl.edu (K.C.); aelchynski@cop.ufl.edu (A.E.); AElsey@cop.ufl.edu (A.R.E.); LCavallari@cop.ufl.edu (L.H.C.); kwiisanen@cop.ufl.edu (K.W.); julie.johnson@ufl.edu (J.A.J.); jgums@UFL.EDU (J.G.G.); 2 Center for Pharmacogenomics and Precision Medicine, College of Pharmacy, University of Florida, 1345 Center Dr, Gainesville, FL 32603, USA; 3 Division of General Internal Medicine, College of Medicine, University of Florida, 1329 SW 16th St, Gainesville, FL 32608, USA; Eric.Rosenberg@medicine.ufl.edu (E.I.R.); Katherine.Huber@medicine.ufl.edu (K.N.H.); Ying.Nagoshi@medicine.ufl.edu (Y.L.N.); Ashleigh.Wright@medicine.ufl.edu (A.W.); Jeffrey.Budd@medicine.ufl.edu (J.T.B.); Neal.Holland@medicine.ufl.edu (N.P.H.); Edlira.Maska@medicine.ufl.edu (E.M.); Danielle.Panna@medicine.ufl.edu (D.P.); 4 Clinical and Translational Science Institute, University of Florida, 2004 Mowry Rd, Gainesville, FL 32610, USA

**Keywords:** precision medicine, pharmacogenetics, pharmacogenomics, implementation, primary care, internal medicine, CYP2C19, CYP2D6

## Abstract

Pharmacogenetic testing (PGT) is increasingly being used as a tool to guide clinical decisions. This article describes the development of an outpatient, pharmacist-led, pharmacogenetics consult clinic within internal medicine, its workflow, and early results, along with successes and challenges. A pharmacogenetics-trained pharmacist encouraged primary care physicians (PCPs) to refer patients who were experiencing side effects/ineffectiveness from certain antidepressants, opioids, and/or proton pump inhibitors. In clinic, the pharmacist confirmed the need for and ordered *CYP2C19* and/or *CYP2D6* testing, provided evidence-based pharmacogenetic recommendations to PCPs, and educated PCPs and patients on the results. Operational and clinical metrics were analyzed. In two years, 91 referred patients were seen in clinic (mean age 57, 67% women, 91% European-American). Of patients who received PGT, 77% had at least one CYP2C19 and/or CYP2D6 phenotype that would make conventional prescribing unfavorable. Recommendations suggested that physicians change a medication/dose for 59% of patients; excluding two patients lost to follow-up, 87% of recommendations were accepted. Challenges included PGT reimbursement and referral maintenance. High frequency of actionable results suggests physician education on who to refer was successful and illustrates the potential to reduce trial-and-error prescribing. High recommendation acceptance rate demonstrates the pharmacist’s effectiveness in providing genotype-guided recommendations, emphasizing a successful pharmacist–physician collaboration.

## 1. Introduction

The implementation of pharmacogenetics has grown in various healthcare settings, because of advancements in technology, accumulation of evidence of genetic associations with drug response, and increased provider and pharmacist training [1,2,3,4,5,6]. The field of pharmacogenetics focuses on the impact of interindividual variability in drug response due to genetic variation and the usage of this information to guide drug therapy decisions; pharmacogenetic test results can be utilized as another tool to individualize drug therapy, similar to monitoring serum creatinine levels or drug interactions [7]. By adjusting medication therapy (i.e., selecting different drug or dose) based on genetic variants and other patient-specific factors, pharmacogenetics aims to reduce toxicity and/or increase effectiveness of medications to further optimize patient care [8].

Thus far, pharmacogenetic implementations have largely taken place in research hospitals [9] or academic medical centers [10,11,12,13,14,15], which often have the financial means and access to trained personnel necessary to facilitate pharmacogenetic testing and its integration into practice. In addition, teaching environments foster interdisciplinary collaborations and engagement with physician champions, two key pieces that have been shown to be essential for longevity of pharmacogenetic services [16]. Nevertheless, pharmacogenetic implementations are not limited to research or academic health systems [17,18,19]. Previous publications have described how to implement pharmacogenetic services in various settings, focusing on required resources and ways to overcome common challenges [16,20,21].

Regardless of setting, it is crucial for those leading pharmacogenetic implementations to understand the evidence supporting genotype-guided therapy. To improve applicability of pharmacogenetic test results, there are evidence-based guidelines available from consortia such as the Clinical Pharmacogenetics Implementation Consortium (CPIC) [22] and Dutch Pharmacogenetics Working Group (DPWG) [23]. Additionally, the Pharmacogenomics Knowledge Base (PharmGKB; available at www.pharmgkb.org) is a resource for clinicians and researchers that contains curated data on the influence of genetic variation on drug response, including pharmacogenetic information within the FDA-approved drug labeling [24]. An evidence-based approach ensures that pharmacogenetic testing is used appropriately to complement the clinical decision-making process, and in doing so, is more likely to lead to improved patient outcomes.

In order to contribute to the evidence base of the real-world utility of clinical implementation of genomic medicine (including pharmacogenetics) and support the development, investigation, and dissemination of genomic medicine practice models, the National Institutes of Health-funded IGNITE (Implementing GeNomics In pracTicE; www.ignite-genomics.org) Network was established in 2013 [25]. One of the projects within this network was led by the University of Florida (UF) Health Precision Medicine Program [14,26], which is a multidisciplinary team of pharmacists, physicians, informaticians, and others who have implemented multiple examples of pharmacogenetic testing into practice and provided evidence-based pharmacogenetic guidance on drug/dose selection. Since it was established in 2011, the Precision Medicine Program has launched six implementations across various practice areas in inpatient and outpatient settings [14,26], largely focusing on *CYP2C19* and *CYP2D6* and applicable medications. However, previous implementations did not offer face-to-face consultation between the patient and pharmacist and were instead focused on specific therapeutic areas or medication classes (e.g., chronic pain and CYP2D6-metabolized opioids [e.g., codeine, tramadol, hydrocodone] or depression and certain selective serotonin reuptake inhibitors [SSRIs; all except fluoxetine]).

It was a logical next step to develop an outpatient clinic where a pharmacogenetics-trained pharmacist could provide guidance to primary care physicians on multiple medications for various disease states based on pharmacogenetic test results. This collaboration between a specialist pharmacist and primary care physicians seemed like a natural fit, as evidence had shown that these physicians were interested in pharmacogenetics but were uncomfortable utilizing these test results in clinical care [27]. On 5 September 2017, the Precision Medicine Program launched a comprehensive, referral-based pharmacogenetics consult clinic within a UF Health internal medicine clinic. The objective of this article is to describe the development of this clinic, its workflow, and early implementation results, along with challenges encountered, successes, and lessons learned during the first two years of implementation.

## 2. Materials and Methods

### 2.1. Clinic Development

Planning for the clinic implementation started about one year prior to launch. Key steps included selecting a practice site, creating a business model, developing the clinic workflow, establishing a collaborative practice agreement with physicians, and educating physicians and support staff.

The pharmacogenetics consult clinic was integrated into the chosen general internal medicine clinic for several reasons. First, an ambulatory care pharmacist already had an established anticoagulation practice at this site that was well received by the clinic physicians; we aimed to build upon that positive, interdisciplinary relationship. Second, several physicians at that site had experience with pharmacogenetic testing through their recent participation in a trial focused around *CYP2D6* testing for opioids in chronic pain patients [28] and were interested in expanding testing for other medications. Third, medication utilization data (described below) collected from the selected primary care clinic was favorable.

Due to the established infrastructure of the Precision Medicine Program, in-house testing for *CYPC19* and *CYP2D6* was already available to guide prescribing of commonly used medications in the primary care setting, such as SSRIs [29], certain opioids (i.e., codeine, tramadol, hydrocodone, and oxycodone) [28,30], and proton pump inhibitors (PPIs) [31,32]. Health system records were queried for the year prior to clinic launch (1 September 2015–31 August 2016) for the number of patients at UF Health primary care clinics prescribed these medications. To aid referral volume, the goal was to identify a primary care clinic that had a large patient population and a high proportion of patients receiving at least one of these target medications.

**Figure 1:**
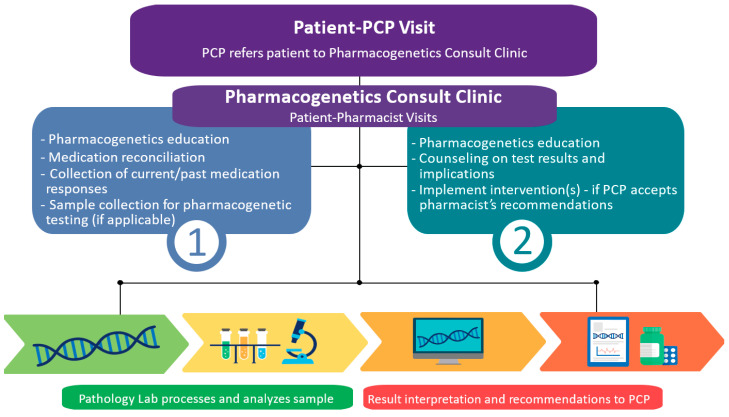
Clinical workflow of the pharmacogenetics consult clinic.

Along with selecting a site to launch the clinic, a clinical workflow was created. At that time, an existing pharmacogenetics consult clinic typically had two visits with the patient [18], and their model was used as a guide when creating the workflow (Figure 1). The team wanted the pharmacist to first meet with patients and complete a detailed medication and medical history to determine if proceeding with pharmacogenetic testing would provide benefit. If testing was not indicated for current medications, this initial visit would provide an opportunity for the pharmacist to counsel the patient and the physician on appropriate use of testing for specific medications, increasing likelihood of reimbursement. After the pharmacogenetic test results were available, a second, dedicated visit was considered essential to counsel the patient on the results and implications for past, current, and/or potential future medications.

Given the pilot nature of clinic, the visits were initially allotted 40 min, allowing up to 6 visits per 4-hour session. Since the eventual goal is to offer this clinic as a revenue-generating service, this visit schedule would allow the pharmacist to be cost neutral at a 0.1 full-time equivalent, based on level 3 Medicare reimbursement estimates and utilizing the general internal medicine physicians as billable providers. Despite having a business plan in place, grant funding was obtained to cover the initial costs of pharmacogenetic testing and the pharmacist’s time in clinic (i.e., 4-hour schedule block once weekly) while workflow procedures were optimized. A collaborative practice agreement was drafted between the pharmacist and the supervising physicians to outline and authorize the clinical services to be provided by the pharmacist, including ordering pharmacogenetic tests.

**Figure 2:**
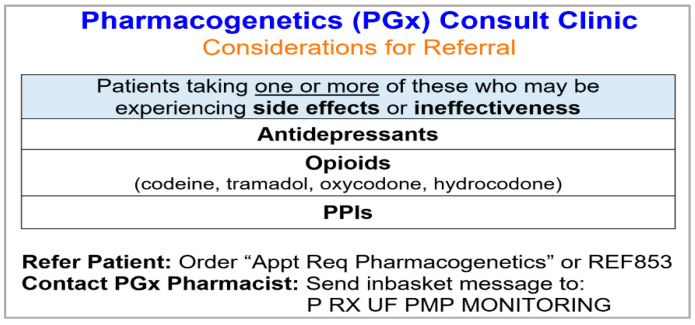
Educational handout provided to physicians with suggested criteria for patient referral.

An additional crucial component of the clinic development was education. Prior to launch, there were several meetings with the medical director and physicians to educate them on the types of patients to refer (Figure 2), how to refer patients within the electronic health record (EHR), the clinic workflow, and to solicit feedback. The pharmacist disseminated small, laminated handouts for physicians with this education. Physicians were educated to refer patients who were experiencing adverse and/or ineffective response to antidepressants, PPIs, and certain opioids (i.e., codeine, tramadol, hydrocodone, and oxycodone). However, physicians were not limited to referring patients only on these medications (e.g., physician could refer patient on clopidogrel) or patients with current medication issues (e.g., physician could be planning to start patient on antidepressant). Additionally, the support staff within the internal medicine clinic were educated on clinic-specific logistics related to scheduling and documentation.

### 2.2. Clinic Workflow

Upon referral by a general internal medicine physician, the clinical pharmacist saw patients in one or two visits, depending on whether a pharmacogenetic test was ordered (Figure 1). During the first visit, the pharmacist educated the patient on pharmacogenetics, discussing key concepts, and tailoring the discussion to the patient’s current and/or past medications with pharmacogenetic implications. An important part of this discussion was the benefits, risks, and limitations of pharmacogenetic testing [33], to ensure that practical expectations were set and that the patient was fully informed before making the decision to undergo testing. Next, the pharmacist took a thorough medication and medical history, with emphasis on current and/or past medications influenced by *CYP2C19* and/or *CYP2D6* testing, including but not limited to SSRIs, PPIs, clopidogrel, CYP2D6-guided opioids (i.e., codeine, tramadol, hydrocodone, oxycodone), and ondansetron. If the pharmacist concluded that pharmacogenetic testing was warranted based on this discussion and the patient was agreeable, the pharmacist collected a buccal sample from the patient for testing at the end of the visit, avoiding any possible delays in sample collection that often occur with off-site collection. Once grant funds for initial pharmacogenetic tests were exhausted in February 2019, the team began billing patients’ insurance for testing. Moving forward, at this part of the visit, the pharmacist always had a conversation with the patient about potential max out-of-pocket cost for pharmacogenetic testing, in the event their insurance provider did not cover the test(s). If the patient was agreeable with this cost, then the sample was collected. In a couple cases due to lower cost or ability to test for additional genes, the patient opted to get pharmacogenetic testing from an external commercial laboratory that met the pharmacist’s criteria for clinical use (e.g., the laboratory was Clinical Laboratory Improvement Amendments (CLIA) certified, the genetic variants tested and allele assignment were consistent with in-house laboratory testing as approved by internal regulatory body, variants tested were representative of the patient population, the laboratory’s methodology was judged to be satisfactory (including testing for *CYP2D6* copy number variation), and raw genotypes were provided). Whereas in a few cases, patients had previous pharmacogenetic test results from a commercial laboratory, which were used to guide treatment decisions without retesting if the laboratory met above criteria.

After the sample was collected, a courier transferred it to the in-house College of American Pathologists (CAP)/CLIA-certified clinical laboratory (UF Health Pathology Laboratories, Gainesville, FL, USA), where it was processed and analyzed for *CYP2C19* and/or *CYP2D6* variants on separate platforms. Panel testing, which included *CYP2C19* and *CYP2D6,* among 7 other pharmacogenes (i.e., GatorPGx panel [34]), became available from the laboratory in July 2019. Information on the laboratory, testing platforms, tested variants, and genotype translation are detailed in Table S1. Once the laboratory uploaded the results into the EHR (typically <1 week later), the pharmacist wrote a note containing an interpretation of the results and any necessary recommendations. The pharmacist developed the recommendations by considering patient-specific factors (e.g., interacting medications, current/past response to medications informed by *CYP2C19*/*CYP2D6*), the reason(s) for referral, and the *CYP2C19* and/or *CYP2D6* result interpretation, with guidance from evidence-based, pharmacogenetic guidelines and primary literature. Interacting medications (e.g., moderate or strong CYP2D6 inhibitors) [35] were an important consideration, as patients’ predicted phenotype could have changed due to these concomitant medications, which could lead to phenoconversion [36,37]. Phenoconversion is a phenomenon by which an individual’s genotype-predicted phenotype is changed into another by an environmental factor like a drug interaction [37]. For example, a patient classified as a CYP2D6 normal metabolizer based on genotype alone can phenoconvert to a poor metabolizer if he/she is taking a strong CYP2D6 inhibitor like bupropion, fluoxetine, or paroxetine [35,36]. The pharmacist approached phenoconversion as previously described [28] with one exception, as detailed in Table S2. The consult note was then routed to the referring physician and if necessary, to specialists managing one or more of the target medications. The pharmacists and physicians discussed the recommendations, which strengthened the collaborative nature of the consult service.

When the patient returned for the second visit (typically 2–4 weeks later based on patient availability and 4-hour once weekly clinic schedule), the pharmacist reiterated important educational concepts from the previous visit and counseled the patient on the pharmacogenetic test results. The pharmacist discussed how the results may impact the patient’s response to current and/or potential future medications, as well as how the results may explain certain responses the patient had to previous medications. Interventions were implemented at this visit if the physician had already accepted the pharmacist’s recommendations. Patients were provided a one-page, double-sided handout with a summary of their results, result interpretation (i.e., phenotype based on genotype alone and drug interactions if applicable), and impacted current and potential future medications. The pharmacist educated the patient on the importance of sharing this document with other healthcare providers, and explained that certain medication additions, discontinuations, or dose changes could alter the interpretation of these results [35,36,37]. In cases of medication changes like these, physicians could refer patients back to the consult clinic for an additional visit to reevaluate the patient’s results with respect to these changes. Due to the importance of face-to-face counseling offered to patients at this second visit, the pharmacist made every effort to see the patient in-person. In extenuating circumstances when the patient was unable to return to clinic, the pharmacist performed the second visit via telephone.

Operational and clinical metrics were collected by the pharmacist for the 2-year timeframe following the launch of the clinic to evaluate the feasibility, sustainability, and clinical usefulness of the service. These metrics are listed and defined in Table 1. Data were collected in accordance with the Declaration of Helsinki with quality improvement project approval by the University of Florida Health Sebastian.

Ferrero Office of Clinical Quality and Patient Safety. Patient characteristics were summarized using descriptive statistics. For patients with pharmacogenetic test results, the frequency of CYP2C19 and CYP2D6 phenotypes (based on genotype alone) were compiled and compared to a reference population of similar ancestry to the majority of the clinic patients [40,41,42,43] (i.e., European) using Fisher’s exact test. *p*-values less than 0.05 were considered significant. Statistical analyses were performed using SAS v. 9.4 (SAS Institute, Cary, NC, USA).

## 3. Results

### 3.1. Medication Utilization

In the 12 months prior to launch of the pharmacogenetics consult clinic, the internal medicine site that was chosen to house this clinic had the largest patient base of all the queried primary care clinics; in this time frame, 9423 unique patients were seen and nearly 60% of patients were taking at least one target medication (Table 2).

### 3.2. Operational Metrics

**Figure 3:**
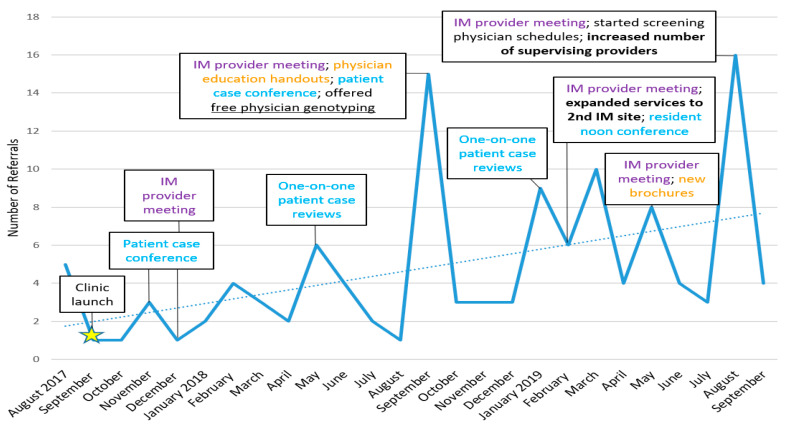
Number of referrals by IM physicians to the pharmacogenetics consult clinic per month, including captions of interventions taken to engage physicians and increase referrals. Similar colored captions indicate similar interventions. Dotted line indicates trend line. IM: Internal Medicine.

**Figure 4:**
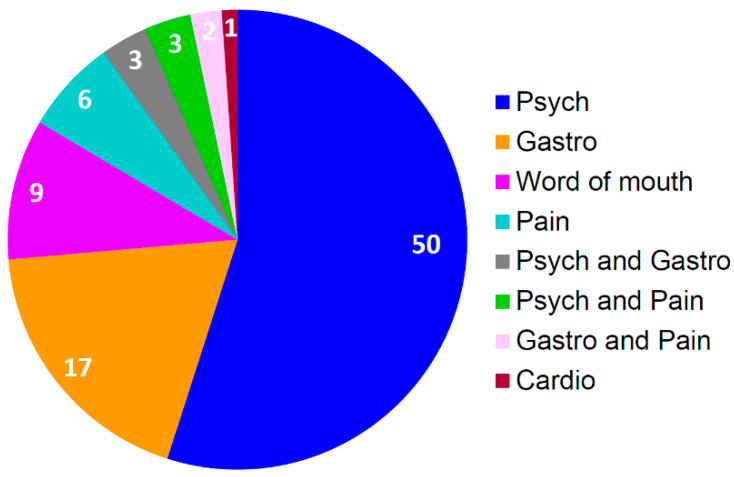
Number of referrals to the pharmacogenetics consult clinic by referral type (i.e., therapeutic area(s) of medication(s) identified by referring internal medicine physician as warranting drug therapy optimization with pharmacogenetics [n = 91], based on suggested referral criteria). Cardio: Cardiology; Gastro: Gastroenterology; Psych: Psychiatry.

In the first two years after clinic launch, 119 patients were referred by general internal medicine physicians to the pharmacogenetics consult clinic (Figure 3), and of these patients, 76% were seen in clinic and 24% were unable to be contacted or decided against scheduling. Physicians referred patients for guidance on medications for psychiatry, gastroenterology, pain, cardiology, and/or combinations of these medications. Over half of the patients seen in clinic were referred based on the use of a psychiatric medication (e.g., SSRI, SNRI), followed by gastrointestinal medication (i.e., PPI) and patient word-of-mouth (Figure 4). For the latter, patients asked their physicians to refer them after learning about clinic via several mechanisms, including the internet (n = 2), family or friends (n = 5), and conferences (n = 2).

**Figure 5:**
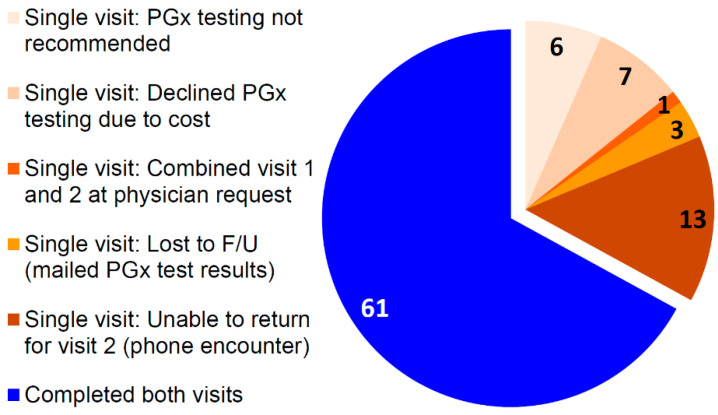
Number of patients completing one (n = 30) or two visits (n = 61) to the pharmacogenetics consult clinic, with reason for completion of single visit. F/U: follow-up; PGx: pharmacogenetic.

Of the 91 patients seen in clinic, one-third had one face-to-face visit with the pharmacist and two-thirds completed both visits (Figure 5). Reasons for only one visit included: pharmacogenetic testing was not recommended, the patient declined testing due to cost, the patient had a combined first and second visit at physician request, the patient was lost to follow-up after their first visit and their results were mailed to them, or patients were unable to return to clinic for their second visit (e.g., scheduling conflicts or transportation challenges) and the visit was conducted over the phone. Typically, first visits lasted 40–60 min, whereas second visits lasted 20–30 min. Interestingly, one patient had a third visit, as a year later they decided to undergo additional pharmacogenetic testing on a psychotropic commercial panel containing pharmacodynamic genes (i.e., genes encoding the serotonin transporter and a serotonin receptor), and they wanted further education on these new results.

**Figure 6:**
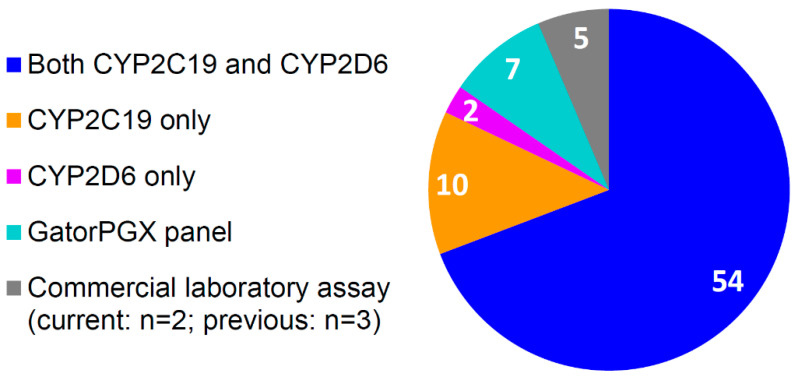
Number of patients (n = 78) with pharmacogenetic test results by gene/assay.

Overall, the pharmacist recommended pharmacogenetic testing for 93% (82/88) of clinic patients; three patients had prior *CYP2C19* and *CYP2D6* testing performed by a commercial laboratory and based on satisfactory evaluation by the pharmacist, testing was not repeated. The pharmacist advised against testing for four patients who were not currently taking medications that could be guided by testing and for two patients who were responding as expected to their medications. Of the patients the pharmacist recommended to undergo testing, 8.5% (7/82) refused due to cost, indicating a patient genotyping acceptance rate of 91.5% (75/82). Including patients with previous test results, seventy-eight patients in total had pharmacogenetic testing, of which, 70% received testing for both *CYP2C19* and *CYP2D6*, 13% for *CYP2C19* only, and 3% for *CYP2D6* only, while 9% underwent testing on the GatorPGx panel, and 6% received testing via commercial laboratory assay (containing *CYP2C19* and *CYP2D6*) (Figure 6). For samples genotyped in the in-house clinical laboratory, the median turnaround time from sample collection to result generation in the EHR was 5 (IQR 3–8) days for *CYP2C19*, 6 (IQR 4–8) days for *CYP2D6*, and 4 (IQR 4–5) days for the GatorPGx panel.

After billing began for pharmacogenetic testing in February 2019 until data was collected through early September 2019, 36 patients were seen in clinic. Excluding two patients who had previous pharmacogenetic test results from an approved commercial laboratory, the pharmacist recommended pharmacogenetic testing for 91% (31/34) of patients, of which, 6% declined in-house testing due to cost and opted for testing from a commercial laboratory approved by the pharmacist (and then returned for a second visit), 23% declined any testing due to cost, and 71% underwent in-house testing.

### 3.3. Clinical Metrics

A total of 91 patients completed the first visit (Table 3). Mean age was 57 years, 67% were female, and 91% were European American. Of patients referred solely for psychiatric medication guidance who received testing, 52% (23/44) were taking at least one other medication that could be impacted by *CYP2C19/CYP2D6* (39% PPI, 18% CYP2D6-guided opioid, 9% ondansetron, 2% clopidogrel). Of patients referred solely for PPI guidance who received testing, one-third (5/15) were taking at least one other genotype-guided medication (20% CYP2C19-guided SSRI, 13% CYP2D6-guided opioid).

Of the patients tested for *CYP2C19* and/or *CYP2D6*, 95% (74/78) were currently taking or planning to take a medication that could be guided by at least one of these genes. Testing was ordered for four patients who were not currently taking any medication with strong pharmacogenetic evidence, but based on their past medical history, there was believed to be a high likelihood of them needing such a medication in the future. As such, the pharmacist provided recommendations solely pertaining to potential future therapies to their referring physicians. Of the 74 patients who were currently prescribed (82%) and/or planning to start a medication (39%) that could be impacted by *CYP2C19* and/or *CYP2D6* (Table 1)*,* there was a total sum of 123 genotype-guided medications (Table S3). On average, each of these patients was prescribed or planning to start 1.7 ± 0.8 genotype-guided medications (range 1 to 4).

In comparison to population values for individuals of European ancestry [38,39,40,41], there was a significant difference in the frequencies of CYP2D6 phenotypes (*P* = 0.02) and trending difference in the frequencies of CYP2C19 phenotypes for clinic patients (*P* = 0.06; Table 4), both based on genotype alone.

Twenty-four percent (16/68) of patients with *CYP2D6* test results had a drug interaction with a moderate and/or strong CYP2D6 inhibitor (duloxetine, n = 3; bupropion, n = 8; paroxetine, n = 1; both duloxetine and bupropion, n = 3; both duloxetine and fluoxetine, n = 1), likely causing phenoconversion. Overall, including CYP2D6 drug interactions, nearly 80% (62/78) of patients had at least one CYP2D6 or CYP2C19 phenotype other than normal metabolizer. When considering the patients’ specific current/planned medications that could be impacted by their CYP2C19/CYP2D6 phenotype results (including drug interactions), 77% (57/74) of patients had at least one actionable phenotype that would make conventional prescribing of genotype-guided medications unfavorable.

The pharmacist made 64 total recommendations to physicians to modify a dose or change a medication for 59% (46/78) of patients (Table S3). Excluding two patients who were lost to follow-up with their physician, recommendations were provided on half of the total genotype-guided medications (Table S3), suggesting that physicians consider starting a new (n = 17) or alternative medication (n = 16), discontinuing a medication (n = 9), and/or making a dose change (increase [n = 13]; decrease [n = 7]). Eighty-seven percent of recommendations (54/62) were accepted.

## 4. Discussion

### 4.1. Successes

Initial data from our clinic suggest that a pharmacist-led pharmacogenetics consult clinic within an internal medicine setting is feasible, as demonstrated by the medication utilization data, types of referrals, high percentage of patients recommended to receive pharmacogenetic testing, high patient acceptance rate of testing, and reasonable test turnaround time. This type of service is also clinically useful, as illustrated by the substantial number of patients taking additional genotype-guided medications than indicated by referral, large percentage of patients taking/planning to take a genotype-guided medication, significantly higher and marginally higher frequencies of *CYP2D6* and *CYP2C19* genotypes (respectively) than expected compared to a reference population, number of patients with relevant drug-drug interactions resulting in probable phenoconversion, high percentage of patients with at least one actionable CYP2C19 or CYP2D6 phenotype, number of recommendations, high recommendation acceptance rate, and number of word-of-mouth referrals.

First, medication utilization data showed that our chosen internal medicine clinic site had a large patient panel (~9500 patients) and high proportion (~60%) of patients receiving at least one target medication (i.e., SSRI, PPI, or CYP2D6-guided opioid [codeine, tramadol, hydrocodone, oxycodone]), indicating feasibility of launching a pharmacogenetics consult clinic at that site. Second, as anticipated based on medication utilization data and physician education on types of patients to refer, most patients (nearly 90%) were referred for guidance on antidepressants, PPIs, and/or opioids. Over half of referrals for clinic patients were related to a psychiatric medication (e.g., SSRI, SNRI) and 10% were referred based on word-of-mouth. Third, the pharmacist recommended pharmacogenetic testing for 93% of clinic patients, highlighting that our method of identifying patients who are suitable for pharmacogenetic testing (based on their experience of side effects or lack of effectiveness while taking certain antidepressants, opioids, and/or PPIs) is effective. Fourth, patients accepted the pharmacist’s recommendation to undergo genotyping at an overall rate of 91% for two years and rate of 77% for the last 6 months when patients’ insurance was billed for testing. Although possible out-of-pocket cost of testing discouraged a quarter of patients from testing in that 6-month time frame, this rate seems reasonable in light of the current reimbursement climate. Still, a large percentage of patients underwent testing, enabling the pharmacist to interpret the results, provide guidance to the physician, and educate the patient on the results face-to-face nearly 80% (62/78) of the time, followed by phone consultation 17% (13/78) of the time. Lastly, our data further suggest that a pharmacist-led pharmacogenetics consult clinic is feasible, determined from the practical testing turnaround time (median 4–6 days), allowing patients to return as soon as two weeks later to receive counseling on their results and potentially have changes made to their medications in effort to optimize their treatment for depression, anxiety, gastroesophageal reflux disease (GERD), and/or chronic pain.

Moving beyond feasibility, our data demonstrates that a pharmacist-led pharmacogenetics consult clinic is clinically useful. A considerable number of patients were taking other genotype-guided medications than indicated by referral, illustrating value added by the pharmacist in identifying additional opportunities to optimize the patient’s medication regimen. One such example involved a patient who was referred to clinic for uncontrolled depression while taking a CYP2C19-guided SSRI; upon medication reconciliation, the pharmacist learned that the patient was taking a CYP2D6-guided opioid as well. Furthermore, the number of patients taking other genotype-guided medications supports the clinical utility of pharmacogene panel-based testing that includes *CYP2C19* and *CYP2D6*. This corroborates the findings from a recent study led by El Rouby and colleagues, which assessed the prevalence of drugs that can be guided by 5 pharmacogenes (i.e., *CYP2C19, CYP2D6, CYP2C9, VKORC1-1639, SLCO1B1*) and opportunities for genotype-guided prescribing among patients with percutaneous coronary intervention [44]. This investigation uncovered a high prevalence of actionable phenotypes in the University of Florida Health cohort and a national cohort of privately insured patients, ultimately supporting the value of panel-based pharmacogenetic testing.

In line with the value of panel-based pharmacogenetic testing, the development and implementation of the GatorPGx panel (Table S1) was successful [34]. Our experience with ordering and utilizing the results has been positive; the panel enables us to provide guidance on additional medications, at a lower out-of-pocket cost to patients compared to the current cost for individual *CYP2C19* and *CYP2D6* testing. Plus, unlike many commercial labs whose pharmacogenetic test results do not interface with our EHR, these results are entered by our lab as discrete (structured) data into the EHR, allowing our program-developed clinical decision support alerts to fire. If/when one of the clinic patient’s physicians orders a medication for a patient whose genotype results place them at risk for adverse effects/ineffectiveness, then a pop-up alert would briefly explain the scope of the problem and provide recommendation(s) to the physician suggesting an alternative drug/dose [10,45].

Another measure of the clinical usefulness of our service was the large percentage (95%) of patients taking/planning to take a medication guided by *CYP2D6* and/or *CYP2C19*. Moreover, based on genotype alone, we observed significantly higher and marginally higher frequencies of *CYP2D6* and *CYP2C19* genotypes, respectively, in clinic patients compared to a European reference population. Before considering CYP2D6 drug interactions, we observed almost two times more CYP2D6 poor metabolizers in clinic compared to the reference population, which would increase at least 2-fold more after considering phenoconversion. Additionally, in clinic we observed 50% more CYP2C19 ultra-rapid metabolizers and almost three times more CYP2C19 poor metabolizers compared to the reference population. Collectively, including drug interactions, we observed a high percentage (77%) of patients with at least one actionable CYP2C19 or CYP2D6 phenotype, which would make usual prescribing of genotype-guided medications unfavorable. Together, the large percentage of patients taking/planning to start a genotype-guided medication, the higher than expected genotype frequencies observed in clinic patients, and the high percentage of patients with at least one actionable phenotype highlights that the general internal medicine providers are correctly identifying patients to refer to clinic based on our suggested referral criteria, emphasizing that our education on who to refer has been successful.

The pharmacist educated physicians primarily on two key aspects: (1) identifying the relevant *CYP2D6* or *CYP2C19* genotype-guided medications that the patient is taking and (2) assessing whether the patient has experienced side effects or ineffectiveness while taking one or more of those medications. These possible medication responses (i.e., toxicity/efficacy issues) are precisely what we would expect to see in patients with actionable phenotypes, such as increased adverse effects in a CYP2C19 poor metabolizer who chronically takes PPIs (e.g., more frequent respiratory infections) or inadequate response to escitalopram in a CYP2C19 ultra-rapid metabolizer. Since over half of our clinic patients were referred for uncontrolled depression and/or anxiety or intolerable adverse effects from certain antidepressants, we often encountered patients who had already tried or failed several antidepressants. The high percentage of patients with at least one actionable phenotype exemplifies the potential of pharmacogenetics to reduce trial-and-error prescribing, an approach that would prove especially beneficial for patients with depression. A meta-analysis of five randomized clinical trials conducted by Bousman et al. showed that patients receiving genotype-guided therapy were 1.71 times more likely to achieve symptom remission compared to patients receiving the usual trial-and-error approach, supporting the utility of pharmacogenetics to guide depression therapy [46].

Other data that reinforces the usefulness of the pharmacogenetics consult clinic includes our observation of drug interactions that could alter patients’ phenotypes in nearly a quarter of patients; this relatively high frequency of drug interactions shows the importance of considering genotype in the context of drug interactions, something that pharmacists are uniquely trained to do, thus emphasizing the value of pharmacists in pharmacogenetics clinics. In addition, the pharmacist made 64 total recommendations to physicians suggesting changing a medication/dose for nearly 60% of clinic patients, which were frequently accepted. This high recommendation acceptance rate suggests that the general internal medicine physicians trust or rely on the expertise of the pharmacist in ordering pharmacogenetic tests when appropriate, interpreting results, and providing sound recommendations. The interdisciplinary collaboration between the pharmacist and primary care physicians has been instrumental to the early success of this clinic.

Lastly, a pharmacist-led pharmacogenetics consult clinic in collaboration with general internal medicine physicians holds the promise to improve patient care with a high level of patient satisfaction. Several patients have told their friends and family about their positive experience, which has contributed to word-of-mouth referrals comprising 10% of referred patients seen in clinic. This organic form of advertising for our service illustrates patient value of receiving pharmacogenetic testing and counseling on the implications of the results. Further, it suggests that this subset of patients who spread the word and those who in turn sought out a referral from their physician are proactive in their healthcare, highlighting the potential of pharmacogenetics as a tool to empower patients to become more proactive in their own healthcare and illustrating the benefit of positive patient testimonials.

### 4.2. Challenges

As anticipated, potential out-of-pocket cost of pharmacogenetic testing was a barrier we encountered once we began billing patients’ insurance for testing. Of patients for whom pharmacogenetic testing was recommended after billing for testing began, 6% declined in-house testing and received testing from a commercial laboratory and nearly a quarter of patients declined any testing due to potential out-of-pocket cost. Reimbursement data by testing indications (e.g., ICD-10 codes for depression, anxiety, GERD) is needed to educate patients on which insurance providers are generally reimbursing for *CYP2C19*, *CYP2D6*, and/or panel testing. Although from our experience, these data cannot always provide answers for patients or their physicians regarding test coverage with 100% certainty. Otherwise, adequate support staff are needed to complete prior authorizations before testing to justify medical necessity and to submit appeals for denied claims in scenarios where testing is deemed warranted (e.g., based on current/past medication response history and past medical history). In some cases where testing was indicated but cost was an issue for the patient, we recommended that they proceed solely with testing for a single gene (e.g., *CYP2C19*) instead of testing on the GatorPGx panel or forgoing testing altogether, based on the likelihood of the patient having an “actionable” result for that gene (e.g., patient with uncontrolled depression was referred to clinic because they failed escitalopram, a CYP2C19-guided SSRI) and cheaper cost.

Lastly, a continual challenge has been maintaining a steady referral rate, which has implications for the sustainability of the clinic. We have learned that the physical presence of the pharmacist in clinic is very important, as this visibility reminds physicians to refer patients and allows for discussion of recent recommendations. However, in order to justify additional clinic sessions to increase the pharmacist’s time in clinic, the referral volume would need to be higher, which presents a dilemma. One strategy that has proven helpful to increase referrals is to conduct periodic education sessions with the physicians, either individually or in group meetings, using that opportunity to review patient cases from clinic. These sessions illustrate the potential clinical value of the service and remind physicians about the types of patients to refer. Immediately after conducting these sessions, there were increases in referrals, although these spikes were not sustained. Similar to another pharmacogenetics clinic during its first two years [18], referrals returned close to baseline after several weeks.

In order to improve sustainability of the clinic, the pharmacogenetics-expert pharmacist and Precision Medicine Program colleagues undertook several key measures, as shown in Figure 3. In an attempt to increase referrals and engage physicians who had not yet referred their patients, we offered physicians free personal pharmacogenetic testing [26,47]. The pharmacist reviewed the results with each physician one-on-one to ensure understanding of these results and implications for their current/potential future medications. The intent of this activity was not only to familiarize them with the process and education that their patients receive upon result interpretation, but to illustrate the clinical relevance and ability of these results to inform commonly prescribed medications. Secondly, one and a half years after clinic launch, we opened up referrals to a second internal medicine clinic that was recently established, which was also directed by our medical director (who was a champion for our service). Additionally, we designed flyers and brochures to display in patient exam rooms, which advertise the availability of the service directly to patients and show common medications that can be impacted by the genes that we are testing. The aim of these flyers and brochures was to encourage dialogue between the patient and their physician about the pharmacogenetics consult clinic, with the goal to increase referrals.

While these advertising resources have been useful, other strategies to increase referral rates appear to have been more effective, including pharmacist attendance at twice monthly provider meetings and expansion of physicians’ role in clinic. First, pharmacist attendance at the provider meetings allowed for brief discussion with the physicians about eligible patients for referral and the pharmacist could follow-up on recommendations before/after the meeting. This meeting also served a serendipitous purpose on a few occasions, as it provided an opportunity for several providers to champion the service, vocalizing their and their patients’ satisfaction/excitement with our service. Second, we expanded physicians’ role in clinic after the service workflow had been optimized with the two initial supervising physicians, by creating a rotating schedule of six interested attending physicians. This schedule was utilized whenever a patient’s primary care physician was not working at the internal medicine clinic at the time of their patient’s visit with the pharmacogenetics pharmacist. Expansion of physicians’ role in clinic was advantageous because it allowed for increased problem solving between pharmacist and physicians, ultimately strengthening the collaboration. One future direction to build upon this productive collaboration could be to integrate the pharmacogenetics service into other conventional pharmacy operations in clinical practice—i.e., this service could become an aspect/component of anticoagulation, deprescribing consultation, prior authorization reviews, and/or collaborative hypertension clinics.

## 5. Conclusions

In our experience, implementation of a pharmacist-led pharmacogenetics consult clinic in collaboration with general internal medicine physicians was shown to be feasible, as demonstrated by medication utilization data, high percentage of patients recommended to receive pharmacogenetic testing, high patient acceptance rate of testing, and test turnaround time, and clinically useful to provide guidance on commonly prescribed medications, as illustrated by the large percentage of patients taking/planning to take a genotype-guided medication, high percentage of patients with at least one actionable CYP2C19 or CYP2D6 phenotype, high recommendation acceptance rate, and number of word-of-mouth referrals. In agreement with other pharmacist-led pharmacogenetics clinics, maintaining constant referral rates is challenging, however, utilizing strategies to increase visibility and dialogue between pharmacist and physicians can serve as a solution to increase referral volume until more consistent approaches/models are identified to achieve sustainability. For the first two years post clinic launch, data is currently being collected from physician notes in the EHR related to clinic patients’ response to the pharmacist’s genotype-guided interventions.

## Figures and Tables

**Table 1:** Operational and clinical metrics collected by the pharmacist on the pharmacogenetics consult clinic.

Operational Metrics	Definitions/Examples/Comments
Number of referrals by general IM physicians	Total, by month
Types of referrals by general IM physicians	Therapeutic area(s) of medication(s) identified by the referring IM physician as warranting drug therapy optimization with PGx (e.g., psychiatry, gastroenterology, pain, or combinations of these areas) Word of mouth (including how patient learned about the service [e.g., family member])
Number of referred patients lost to F/U, with reason	Example reasons: patient was unable to be contacted, patient decided against scheduling (e.g., cost, time, transportation)
Number of patients completing one or two visits, including reason for completion of single visit	Example reasons for completion of only single visit: PGx testing was not recommended by pharmacist Patient declined PGx testing (e.g., due to cost) Patient was unable to return for visit 2, therefore pharmacist emailed them the PGx test result handout and conducted a telephone encounter
Visit length	Approximate, in minutes
Number of patients recommended to receive and advised against PGx testing (with reason) ^1^	Example reasons why patients were advised against testing: patient was responding appropriately to or not currently taking medications with *CYP2C19/CYP2D6* evidence
Number of patients with PGx tests ordered ^1^, including test type	Types: name of gene or assay of PGx test (e.g., *CYP2C19, CYP2D6, CYP2C19* and *CYP2D6,* GatorPGx panel [i.e., 9 pharmacogene panel offered by internal lab, Table S1])
Number of patients who refused PGx testing, including reason ^1^	Example reasons: cost, privacy concerns, unsure of value
Genotyping acceptance rate by patient	= $\frac{\mathrm{Number}\;\mathrm{of}\;\mathrm{patients}\;\mathrm{with}\;\mathrm{PGx}\;\mathrm{tests}\;\mathrm{ordered}}{\mathrm{Number}\;\mathrm{of}\;\mathrm{patients}\;\mathrm{with}\;\mathrm{PGx}\;\mathrm{tests}\;\mathrm{ordered}+\mathrm{Number}\;\mathrm{of}\;\mathrm{patients}\;\mathrm{refusing}\;\mathrm{PGx}\;\mathrm{testing}}$
Number of patients with previously ordered PGx testing ^1^	Included whether the PGx test met internal established criteria (described in text above) or whether the patient had to repeat testing
PGx test turnaround time	Time between sample collection and result being placed in the electronic health record
**Clinical Metrics**	**Definitions/Examples/Comments**
Patient demographics	Age, sex, race/ethnicity
Number of patients referred for guidance on one medication but pharmacist identified other medications that could potentially be impacted by *CYP2C19/CYP2D6*	Example: Of the patients referred solely for psychiatric medication guidance, X% were taking at least one other medication that could be impacted by *CYP2C19* or *CYP2D6* (X% PPI, X% CYP2D6-guided opioid, X% clopidogrel). Included drug class of other medications identified, unless gene-drug effect applied to single drug; excluded patients w/o PGx testing
Pharmacogenetic test results	*CYP2C19* and/or *CYP2D6* genotype and phenotype per lab; determined predicted phenotype based on drug interactions (Table S2)
Number of patients on moderate and/or strong CYP2D6 inhibitor [35]	Moderate CYP2D6 inhibitor: duloxetine, mirabegron Strong CYP2D6 inhibitor: bupropion, paroxetine, fluoxetine
Number of patients taking/planning to take ^2^ genotype-guided medication	Genotype-guided medications: Current/ planned ^2^ medications that could be impacted by *CYP2C19* and/or *CYP2D6* per CPIC and/or DPWG guidelines [23,29,30,38,39]: SSRIs except fluoxetine, venlafaxine, aripiprazole, PPIs, certain opioids (i.e., codeine, tramadol, hydrocodone, oxycodone), clopidogrel, ondansetron Summarized descriptive statistics for medications, excluding patients w/o testing
Number and names of genotype-guided medications (visit 1)	
Number of patients with at least one actionable phenotype	Actionable phenotype: Phenotype warranting change in prescribing, dependent on gene-drug pair, as defined by CPIC and/or DPWG [23,29,30,38,39]
Number of patients with a recommendation to modify a dose or change a medication	Included recommendations pertaining to genotype-guided medications and medications relating to referral type (e.g., H2 receptor antagonist for patient referred for uncontrolled GERD/ lack of PPI effectiveness)
Number/type of recommendations	Type: New medication, alternative medication, discontinue medication, dose change ↑↓
Recommendation acceptance rate	= $\frac{\mathrm{Number}\;\mathrm{of}\;\mathrm{recommendations}\;\mathrm{accepted}\;\mathrm{by}\;\mathrm{the}\;\mathrm{physician}}{\mathrm{Number}\;\mathrm{of}\;\mathrm{recommendations}\;\mathrm{provided}\;\mathrm{to}\;\mathrm{the}\;\mathrm{physician}}$ Recommendations were considered to be accepted if there was a dosage or drug therapy change made consistent with the recommendation and (1) documentation within the EHR acknowledging the recommendation or (2) in-person/ telephone/ electronic confirmation of recommendation acceptance with the physician. Excluded recommendations if patient was lost to F/U with physician after PGx visit

CPIC: Clinical Pharmacogenetics Implementation Consortium; DPWG: Dutch Pharmacogenetics Working Group; EHR: electronic health record; F/U: follow-up; GERD: gastroesophageal reflux disease; IM: internal medicine; PGx: pharmacogenetic; PPI: proton pump inhibitor; SSRI: selective serotonin reuptake inhibitor; w/o: without. ^1^ These metrics were also summarized separately once the clinic began billing for PGx testing. ^2^ Planned medication: Medication that the patient is not currently taking but their physician is considering having them start or switch to this medication (e.g., patient may be treatment naïve to genotype-guided medication class or may have had history of adverse drug reaction and/or lack of effectiveness with past use of this genotype-guided medication class).

**Table 2:** Medication utilization at selected internal medicine site. ^1.^

CYP2D6-Guided Opioid (i.e., Codeine, Tramadol, Hydrocodone, Oxycodone)	SSRI	PPI	Any of These Medications
4015 (42.6%)	1955 (20.7%)	2985 (31.7%)	5445 (57.8%)

^1^ Visits: 1 September 2015–31 August 2016; out of 9423 total patients seen at this site during this period. PPI: proton pump inhibitor; SSRI: selective serotonin reuptake inhibitor.

**Table 3:** Characteristics of the pharmacogenetics consult clinic patients.

Characteristics	N = 91
Age, years	57 ± 18
Sex, female	61 (67.0)
Race/ethnicity	
European American	83 (91.2)
African American	3 (3.3)
LatinX	2 (2.2)
Unspecified	2 (2.2)
Native Hawaiian/Pacific Islander	1 (1.1)

Data are displayed as mean ± standard deviation or n (%).

**Table 4:** Frequency of CYP2C19 and CYP2D6 phenotypes in the pharmacogenetics consult clinic patients compared to a European reference population. ^1.^

**CYP2C19 Phenotype**	**PGx Clinic Patients ^2^ (n = 76)**	**European Reference Population [40,42,43]**
UM	6 (7.9)	4.7
RM	22 (28.9)	27.2
NM	23 (30.3)	39.6
IM	20 (26.3)	26.0
PM	5 (6.6)	2.4
**CYP2D6 Phenotype**	**PGx Clinic Patients ^3^ (n = 68)**	**European Reference Population** [41,42,43]
UM	2 (2.9)	3.3
NM-UM	3 (4.4)	1.1
NM	48 (70.6)	74.9
IM	6 (8.8)	7.2
PM	8 (11.8)	6.1
Indeterminate	1 (1.5)	7.4

Data are displayed as n (%) or %. UM: ultra-rapid metabolizer; NM-UM: normal to ultra-rapid metabolizer; RM: rapid metabolizer; NM: normal metabolizer; IM: intermediate metabolizer; PM: poor metabolizer; PGx: pharmacogenetics; N/A: not applicable. ^1^ Fisher’s exact test comparisons were made for genotype-derived phenotypes between clinic patients and a reference population derived from Europe. ^2^ Fisher’s exact test, *P* = 0.06. ^3^ Fisher’s exact test, *P* = 0.02.

## Supplementary Materials

The following are available online at https://www.mdpi.com/2077-0383/9/7/2274/s1, Table S1: Pharmacogenetic testing processes, Table S2: CYP2D6 phenotype translation, Table S3: Patients’ current and planned medications that could be guided by *CYP2C19* and *CYP2D6* testing, along with number of recommendations suggesting that physicians consider a change in medication/dose and number of accepted recommendations.
