# Supplementary material for: Design and Early Implementation Successes and Challenges of a Pharmacogenetics Consult Clinic
Source: J Clin Med. 2020 Jul 17;9(7):2274. doi: 10.3390/jcm9072274 (PMC7408871; doi:10.3390/jcm9072274)
Supplement: Supplementary file 1 [file jcm-09-02274-s001.pdf]

**Table S1:** Pharmacogenetic testing processes.

| | Testing Platform | Gene Tested | Alleles Tested[4] |
|---|---|---|---|
| **UF Health Pathology Laboratory**[1] | Luminex xTAG *CYP2D6* Kit v3<br><br>(Luminex Corporation, Austin, TX) | *CYP2D6* [3] | **2, *3, *4, *5, *6, *7, *8, *9, *10 *11, *15, *17, *29, *35, *41, CYP2D6* gene rearrangements associated with the deletion (**5*) and duplication genotypes |
| | eSensor XT-8<br><br>(GenMark Diagnostics, Carlsbad, CA) | *CYP2C19* [3] | **2, *3, *4, *5, *6, *7, *8, *9, *10, *13, *17* |
| | <u>GatorPGx panel</u> [2]<br><br>QuantStudio 12K Flex Real Time PCR System<br><br>(Applied Biosystems by Life Technologies, Carlsbad, CA) | *CYP2D6* | **2, *2A, *3, *4, *5, *6, *7, *8, *9, *10, *17, *29, *41,* rs1135840, *CYP2D6* gene rearrangements associated with the deletion (*5) and duplication genotypes |
| | | *CYP2C19* | *2, *3, *4, *6, *8, *10, *17* |
| | | *CYP3A5* | **3, *6, *7* |
| | | *CYP2C9* | **2, *3, *5, *6, *8, *11* |
| | | *CYP2C9* cluster | rs12777823 |
| | | *CYP4F2* | V433M |
| | | *VKORC1* | c.-1639G>A |
| | | *TPMT* | **2, *3A, *3B, *3C* |
| | | *NUDT15* | **3* |
| | | *SLCO1B1* | **5* |

1 College of American Pathologists-accredited and Clinical Laboratory Improvement Amendments-licensed (CAP/CLIA) clinical laboratory in Gainesville, Florida.

2 The pharmacist collected buccal samples from the patient for testing on the QuantStudio platform. GatorPGx panel was launched in July 2019.

3 Upon clinic launch in September 2017, an offsite phlebotomist collected blood samples from the patient for *CYP2D6* and *CYP2C19* testing on the Luminex and GenMark platforms, respectively. At this time, the Luminex platform was validated for blood and buccal samples for *CYP2D6* testing, however, the GenMark platform was only validated for blood sample collection for *CYP2C19* testing. Once the GenMark platform was validated for buccal samples in October 2017, the pharmacist began collecting buccal brush samples from the patient during the first visit for both *CYP2C19* and *CYP2D6* testing.

4 *CYP2C19* and *CYP2D6* genotypes were translated into phenotypes based on guidance at the time from the Clinical Pharmacogenetics Implementation Consortium (CPIC).[1]

**Table S2:** CYP2D6 phenotype translation.[2,3]

| | |
|---|---|
| **Step 1:** Determined patient's activity score from *CYP2D6* genotype by determining the activity score of each allele present and adding the score together. In the case where a duplication was present, the total score was calculated twice, once with one allele duplicated and once with the other allele duplicated to account for both possibilities (resulting in a ranged phenotype).[1] | |
| **Allele** | **Activity Score** |
| **3, *4, *5, *6, *7, *8, *11, *15* | 0 |
| **9, *10, *17, *29, *41* | 0.5 |
| **1, *2, *35* | 1 |
| **Step 2:** Determined if patient was taking a CYP2D6 inhibitor [2,4], and multiplied the activity score calculated from step 1 by the applicable factor shown below. If patient was prescribed multiple inhibitors, the strongest inhibitor was used in this calculation. | |
| **CYP2D6 inhibitor** | **Multiple activity score by:** |
| Strong inhibitor[2] | 0 |
| Moderate inhibitor | 0.5 |
| Weak inhibitor | N/A |
| **Step 3:** Determined patient's phenotype from the calculated activity score from step 2 as shown below. | |
| **Calculated Activity Score** | **CYP2D6 Phenotype[3]** |
| >2 | UM |
| 1->2 | NM-UM |
| 1-2 | NM |
| 0.25-0.75 | IM |
| 0.5-2 | IM-NM |
| 0 | PM |

UM: ultra-rapid metabolizer; NM-UM: normal to ultra-rapid metabolizer; NM: normal metabolizer; IM: intermediate metabolizer; IM-NM: intermediate to normal metabolizer; PM: poor metabolizer.

[1] Ranged CYP2D6 phenotypes were possible because the assay used did not indicate the exact number of alleles present or which allele was duplicated. For instance, *CYP2D6*1/*4* duplication would translate to CYP2D6 normal to ultra-rapid metabolizer (NM-UM), because this genotype could result in a NM phenotype if the patient's **41* allele was duplicated and a UM phenotype if the patient's **1* allele was duplicated. Patients with a CYP2D6 NM-UM phenotype were treated conservatively as possible UM.

[2] Based on information the pharmacist collected from the patient on their history of response to current and/or past CYP2D6 substrates, the above translation method was not used if the patient with a total activity score >2 was taking a strong inhibitor and appeared to have a predicted phenotype other than PM (in light of being unable to determine exact number of *CYP2D6* copies). In this scenario, the pharmacist instead estimated the CYP2D6 phenotype based on clinical picture, assuming that patient did not undergo complete phenoconversion to PM. For example, if a patient who was taking bupropion (strong CYP2D6 inhibitor) and hydrocodone/ acetaminophen

(CYP2D6 substrate) reported having an exaggerated response to hydrocodone (e.g., severe opioid-induced pruritis and 50% reduction in pain) instead of the expected lack of pain relief common with PM, the pharmacist would estimate a phenotype of CYP2D6 NM-UM for the patient in this case. If CYP2D6 phenotype prediction was not possible based on patient's clinical picture, the phenotype was denoted as "Indeterminate".

[3] This *CYP2D6* genotype to phenotype translation represents the guidance at the time (i.e., September 2017-2019) from CPIC [1], prior to their translation standardization with the Dutch Pharmacogenetics Working Group (DPWG) [5].

**Table S3:** Patients' current and planned medications[1] that could be guided by *CYP2C19* and *CYP2D6* testing, along with number of recommendations suggesting that physicians consider a change in medication/dose and number of accepted recommendations.

| Current/ Planned Medications[1] | Medication Class | Medications | Number of genotype-guided[1] medications (n=123) | Number of recommendations[2] (n=62) | Number of accepted recommendations (n=54) |
|---|---|---|---|---|---|
| Current | **Medications impacted by *CYP2C19*** | | | | |
| | SSRI | citalopram | 1 | 0 | N/A |
| | | escitalopram | 7 | 2 | 2 |
| | | sertraline | 10 | 5 | 4 |
| | PPI | dexlansoprazole | 1 | 0 | N/A |
| | | esomeprazole | 3 | 4 | 4 |
| | | omeprazole | 22 | 11 | 9 |
| | | pantoprazole | 15 | 10 | 8 |
| | | rabeprazole | 1 | 0 | N/A |
| | Antiplatelet | clopidogrel | 1 | 0 | N/A |
| | **Medications impacted by *CYP2D6*** | | | | |
| | Antipsychotic | aripiprazole | 2 | 1 | 1 |
| | SNRI | venlafaxine | 7 | 2 | 2 |
| | SSRI | paroxetine | 1 | 1 | 1 |
| | Opioid | tramadol | 7 | 2 | 2 |
| | | hydrocodone/ APAP | 2 | 2 | 2 |
| | | hydrocodone/ homatropine | 1 | 0 | N/A |
| | | oxycodone (IR/ER) | 4 | 1 | 1 |
| | | oxycodone/ acetaminophen | 3 | 1 | 0 |
| | 5HT3 Receptor Antagonist | ondansetron | 6 | 0 | N/A |
| Planned | **Medications impacted by *CYP2C19* or *CYP2D6*** | | | | |
| | Antidepressant/ Antipsychotic | — | 28 | 20 | 18 |
| | PPI | — | 1 | 0 | N/A |

5HT3: serotonin type 3; PGx: pharmacogenetic; PPI proton pump inhibitor; SNRI serotonin-norepinephrine reuptake inhibitor; SSRI: selective serotonin reuptake inhibitor.

[1] Current/ planned medications that could be impacted by *CYP2C19* and/or *CYP2D6* per CPIC and/or DPWG guidelines [3,6-9]: SSRIs except fluoxetine, venlafaxine, aripiprazole, PPIs, opioids (i.e., codeine, tramadol, hydrocodone, oxycodone), clopidogrel, ondansetron.
[2] Excluded 2 patients with recommendations who were lost to follow-up with their physician.

## References


1. Relling, M.V.; Klein, T.E. CPIC: Clinical Pharmacogenetics Implementation Consortium of the Pharmacogenomics Research Network. *Clinical pharmacology and therapeutics* **2011**, *89*, 464-467, doi:10.1038/clpt.2010.279.
2. U.S. Food and Drug Administration. Drug Development and Drug Interactions: Table of Substrates, Inhibitors and Inducers. Available online: http://www.fda.gov/Drugs/DevelopmentApprovalProcess/DevelopmentResources/DrugInteractionsLabeling/ucm093664.htm (accessed on 1 December 2019).
3. Crews, K.R.; Gaedigk, A.; Dunnenberger, H.M.; Leeder, J.S.; Klein, T.E.; Caudle, K.E.; Haidar, C.E.; Shen, D.D.; Callaghan, J.T.; Sadhasivam, S., et al. Clinical Pharmacogenetics Implementation Consortium guidelines for cytochrome P450 2D6 genotype and codeine therapy: 2014 update. *Clinical pharmacology and therapeutics* **2014**, *95*, 376-382, doi:10.1038/clpt.2013.254.
4. Storelli, F.; Matthey, A.; Lenglet, S.; Thomas, A.; Desmeules, J.; Daali, Y. Impact of CYP2D6 Functional Allelic Variations on Phenoconversion and Drug-Drug Interactions. *Clinical pharmacology and therapeutics* **2018**, *104*, 148-157, doi:10.1002/cpt.889.
5. Caudle, K.E.; Sangkuhl, K.; Whirl-Carrillo, M.; Swen, J.J.; Haidar, C.E.; Klein, T.E.; Gammal, R.S.; Relling, M.V.; Scott, S.A.; Hertz, D.L., et al. Standardizing CYP2D6 Genotype to Phenotype Translation: Consensus Recommendations from the Clinical Pharmacogenetics Implementation Consortium and Dutch Pharmacogenetics Working Group. *Clin Transl Sci* **2020**, *13*, 116-124, doi:10.1111/cts.12692.
6. Hicks, J.K.; Bishop, J.R.; Sangkuhl, K.; Muller, D.J.; Ji, Y.; Leckband, S.G.; Leeder, J.S.; Graham, R.L.; Chiulli, D.L.; A, L.L., et al. Clinical Pharmacogenetics Implementation Consortium (CPIC) Guideline for CYP2D6 and CYP2C19 Genotypes and Dosing of Selective Serotonin Reuptake Inhibitors. *Clinical pharmacology and therapeutics* **2015**, *98*, 127-134, doi:10.1002/cpt.147.
7. Swen, J.J.; Nijenhuis, M.; de Boer, A.; Grandia, L.; Maitland-van der Zee, A.H.; Mulder, H.; Rongen, G.A.; van Schaik, R.H.; Schalekamp, T.; Touw, D.J., et al. Pharmacogenetics: from bench to byte--an update of guidelines. *Clinical pharmacology and therapeutics* **2011**, *89*, 662-673, doi:10.1038/clpt.2011.34.
8. Scott, S.A.; Sangkuhl, K.; Stein, C.M.; Hulot, J.S.; Mega, J.L.; Roden, D.M.; Klein, T.E.; Sabatine, M.S.; Johnson, J.A.; Shuldiner, A.R. Clinical Pharmacogenetics Implementation Consortium guidelines for CYP2C19 genotype and clopidogrel therapy: 2013 update. *Clinical pharmacology and therapeutics* **2013**, *94*, 317-323, doi:10.1038/clpt.2013.105.

9. Bell, G.C.; Caudle, K.E.; Whirl-Carrillo, M.; Gordon, R.J.; Hikino, K.; Prows, C.A.; Gaedigk, A.; Agundez, J.; Sadhasivam, S.; Klein, T.E., et al. Clinical Pharmacogenetics Implementation Consortium (CPIC) guideline for CYP2D6 genotype and use of ondansetron and tropisetron. *Clinical pharmacology and therapeutics* **2017**, *102*, 213-218, doi:10.1002/cpt.598.
